# Suppression of non-small cell lung cancer migration and invasion by hsa-miR-486-5p via the TGF-β/SMAD2 signaling pathway

**DOI:** 10.7150/jca.35017

**Published:** 2019-10-15

**Authors:** Tao Chen, Jianjie Zhu, Tingting Cai, Wenwen Du, Yang Zhang, Qingqing Zhu, Zeyi Liu, Jian-an Huang

**Affiliations:** 1 Department of Respiratory Medicine, the First Affiliated Hospital of Soochow University, Suzhou 215006, China; 2 Suzhou Key Laboratory for Respiratory Diseases, Suzhou 215006, China; 3 Institute of Respiratory Diseases, Soochow University, Suzhou 215006, China

## Abstract

Non-small cell lung cancer (NSCLC) is the leading cause of cancer-related death worldwide. SMAD family member 2 (SMAD2) is a key element downstream of the transforming growth factor beta (TGF-β) signaling pathway that regulates cancer metastasis by promoting the epithelial-mesenchyme transition (EMT). MicroRNA miR-486-5p is a tumor suppressor in NSCLC progression. However, it remains unclear whether miR-486-5p is implicated in TGF-β signaling and EMT in NSCLC. In the present study, high expression of SMAD2 mRNA was detected in NSCLC tissues and cell lines, and was associated with poor survival of patients with NSCLC. By contrast, miR-486-5p was downregulated in NSCLC tissues and cell lines. *In silico* prediction showed that SMAD2 was a potential target of miR-486-5p. The prediction was verified using a dual-luciferase reporter assay. Transwell assays showed that knockdown of SMAD2 inhibited TGF-β-induced EMT and migration and invasion in NSCLC cells. Similarly, miR-486-5p overexpression suppressed TGF-β-induced EMT and migration and invasion of NSCLC cells. The present study provides a new insight into the role of miR-486-5p in regulating TGF-β-mediated EMT and invasion in NSCLC.

## Introduction

Non-small cell lung cancer (NSCLC) is the leading cause of cancer-related death worldwide 1, 2. Although a variety of studies have been conducted on NSCLC cell proliferation, migration, and invasion, the mechanism of NSCLC progression remains unclear and the five-year survival rate of patients with NSCLC remains below 15% 3. More than 90% of deaths from solid tumors, including NSCLC, are mainly attributed to metastasis 4. Hence, it is important to have a good understanding of the mechanisms underlying NSCLC metastasis.

Cell motility is important for tumor cell metastatic dissemination from the primary location to lymph or blood vessels. Transforming growth factor-β (TGF-β) is a cytokine with multiple functions, such as in cell proliferation, differentiation, apoptosis, and cell motility 5, 6. In particular, TGF-β-mediated signaling is markedly altered in NSCLC, and is functionally associated with the tumorigenic and metastatic processes of this disease 7,8. Notably, accumulating evidence suggests that TGF-β signaling is a potent inducer of the epithelial-mesenchymal transition (EMT) in various cancers, including NSCLC 9-11. EMT is vital for morphogenesis during embryonic development and for the conversion of early-stage tumors into invasive malignancies 12,13, which is marked by the repression of E-cadherin expression and the induction of N-cadherin, Vimentin, and Snail expression 11,14. SMAD family member 2 (SMAD2) has been identified as a key element downstream of the TGF-β signaling pathway in regulating cancer metastasis through promoting EMT 15.

MicroRNAs (miRNAs) are a class of small noncoding RNAs that play essential roles in tumor development and progression via the regulation of various signaling networks associated with multiple cellular functions, such as cell proliferation and invasion 16-19. Growing evidence from comprehensive expression analyses shows that many miRNAs are closely related to the development of human lung cancer 20, 21. In our previous analysis of miRNA arrays we found that the expression of miR-486-5p was significantly downregulated in NSCLC tissues 22.

In the present study, to identify new targets of miR-486-5p that may play a role in NSCLC, we predicted its target mRNAs using computational algorithms. Interestingly, the 3'-UTR of *SMAD2* mRNA is a direct target of miR-486-5p. Furthermore, we found that ectopic application of miR-486-5p could inhibit TGF-β-induced EMT and invasion of NSCLC cells. We also discovered a novel mechanism and target of miR-486-5p in NSCLC. MiR-486-5p that directly suppressed *SMAD2* expression, and overexpression of miR-486-5p inhibited the migratory ability of NSCLC cells. Our study provides a new insight into miR-486-5p's regulation of metastasis in NSCLC.

## Materials and Methods

### Tissue samples

Paired NSCLC tissue and adjacent noncancerous lung tissue samples (65 of each) were collected with the informed consent of the patients from the First Affiliated Hospital of Soochow University between 2012 and 2015. The patients had been diagnosed with NSCLC based on their histological and pathological characteristics according to the Revised International System for Staging Lung Cancer. They had not undergone chemotherapy or radiotherapy prior to tissue sampling. The tissue samples were snap frozen and stored in a cryofreezer at - 80 °C. This study was approved by the Ethics Committee of the First Affiliated Hospital of Soochow University.

### Cell Lines and Culture and Transfection

Six human NSCLC cell lines A549, H1299, SPC-A1, H1650, H460 and H226, and one normal human bronchial epithelial cell BEAS-2B were obtained from the Cell Bank of the Chinese Academy of Sciences (Shanghai, China). The cells were grown in Roswell Park Memorial Institute (RPMI) 1640 (HyClone, South Logan, UT)medium containing 10% fetal bovine serum (FBS) (Gibco, Carlsbad, CA, USA) , L-glutamine and penicillin-streptomycin (Invitrogen, Carlsbad, CA, USA) at 37 °C in a humidified atmosphere containing 5% CO_2_.

miR-486-5p mimics, miR-486-5p inhibitor and negative control mimics, inhibitor were obtained from GenePharma (Shanghai, China). The SMAD2 siRNAs and negative control siRNA were designed by Ribobio (Guangzhou, China) , the sequences were as flows siRNA-1: GGUGAAGAAGCUAAAGAAATT, siRNA-2: CAGGCCUUUACAGCUUCUCTT. The mimics, inhibitor or siRNA were transfected into A549 and H226 cells by Lipofectamine^TM^ 2000 reagent (Invitrogen, CA) according to the manufacturers' protocols.

### RNA Extraction, cDNA Synthesis, and Quantitative Real-time PCR (qRT-PCR)

Total RNA wasextracted from cells and tissues using RNAiso Plus reagent was extracted (Takara, Osaka, Japan) according to the manufacturer's protocol. The RNA concentration was measured using a NanoDrop 2000 instrument (Thermo Fisher Scientific, Waltham, MA, USA). Synthesis of cDNA was performed using Reverse Transcriptase M-MLV (Takara). The primers for reverse transcription and amplification of miR-486-5p and U6 were designed and synthesized by Guangzhou RiboBioCorp (Guangzhou, China). The sequences of the primers for qRT-PCR of SMAD2 and ACTB (β-actin) were as follows: SMAD2 Forward: 5'-CAGCCATC-GTTGTCCACT-3', Reverse: 5'-GCTGGGGTGCTGTATGTC-3', β-actin mRNA, Forward: 5'-CACAGAGCCTCGCCTTTGCC-3', Reverse: 5'-ACCCATGCCCACCATCACG-3'. the primers for U6 was purchased from RiboBioCo. Ltd. (Guangzhou, China). qRT-PCR was performed using SYBR Premix ExTaq™ (Takara)according to the manufacturer's instructions on an ABI Step One Plus Real-Time PCR system (Applied Biosystems). The expression values of *SMAD2* mRNA and miR-486-5p were normalized to internal controls *ATCB* and U6, respectively. Relative expression was calculated using the ^ΔΔ^Ct method 23.

### Western Blotting Assay

Cells were grown to 80-90% confluence and then lysed in RIPA buffer (Cell Signaling Technology, Danvers, MA, USA) with protease inhibitors and a phosphatase inhibitor cocktail (Sigma-Aldrich, St. Louis, MO, USA). The samples were centrifuged at 12,000 g for 15 min, and the total cell lysates were separated by 10% SDS-PAGE electrophoresis and transferred to nitrocellulose membranes (Millipore, Billerica, MA, USA). The membranes were blocked with 5% bovine serum albumin (BSA) in Tris-buffered saline-0.1% Tween-20 (TBST) buffer for 1 h at room temperature, incubated with primary antibodies overnight at 4 °C, and then incubated with the corresponding horseradish peroxidase (HRP)-conjugated secondary antibodies for 2 h at room temperature. Detection was performed using an ECL kit (Pierce, Rockford, IL, USA). The band density was quantified using Quantity One 4.6 software. The antibodies used for western blotting , including anti-Smad2, anti-pSmad2, Snail, MMP2, anti-β-actin, anti-mouse and anti-goat secondary antibodies were purchased from Cell Signaling Technology. Antibodies targeting E-Cadherin, N-Cadherin, Vimentin were obtained from BD Biosciences.

### Plasmid Construction, Transient Transfection, and Luciferase Assay

A 225-bp fragment of the SMAD2 3'-UTR containing the miR-486-5p target sites (positions 304-310), as predicted by TargetScan (http://www.targetscan.org/vert_72/) or mutated sites, was synthesized and fused to the 3'-end of a psiCHECK2 dual-luciferase reporter vector (Promega, Madison, WI, USA). A549 and H226 cells were plated in a 24-well plate. The constructed reporter plasmids were co-transfected with either miR-486-5p mimics or a negative control (miR-NC) into the cells using Lipofectamine 2000 (Life Technologies, Carlsbad, CA, USA). The plates were maintained for 48 h, and then the cells were collected and the luciferase activity was measured using a Dual-Luciferase Reporter Assay kit (Promega). Each experiment was conducted in triplicate.

### Wound Healing Assay

A wound healing assay was performed as described previously 24. Briefly, A549 and H226 cells were seeded into 6-well tissue culture plates after 24 h of transfection and cultured until they reached ~80-90% confluence as a monolayer. The monolayer was gently and slowly scratched using a fresh 10-µl pipette tip across the center of the well. The resulting gap distance should equal the outer diameter of the end of the tip. Another scratch was made perpendicular to the first to create a cross in each well. The cells were then washed gently twice with 1 × PBS to remove the detached cells. The well was replenished with fresh medium and the cells were grown for an additional 24 h. The cells were observed and photographed under a microscope(CKX41, Olympus) at the same distance and settings. The width of the gap was evaluated quantitatively using Photoshop.

### Migration and Invasion Assays

Transwell migration and invasion assays were performed as described previously 24. Briefly, 5 × 10^4^ A549 and H226 cells transfected with miRNAs or short interfering RNAs (siRNA) were added to the upper chamber of Transwell plates (BD Biosciences, San Jose, CA, USA) in the presence of 1% FBS in medium. At the same time, 10% FBS medium was added as a chemoattractant to each lower chamber. If necessary, TGF-β1 (5 ng/mL) was added to the upper chamber 6 h later. Cells were allowed to migrate through an 8-μm pore membrane or invade a membrane coated with Matrigel. After 24 h of incubation, the cells that had migrated onto the lower surface of the chamber were fixed with 100% methanol and stained with 1% crystal violet. Finally, the cells were counted in least three random fields under a light microscope.

### Statistical Analysis

An unpaired *t* test (two-tailed) was used to analyze the significance of the data from the cells. A paired *t* test (two-tailed) was performed to determine the significance of the data from patient samples. *P* < 0.05 was considered a significant difference. Statistical analyses were conducted using GraphPad Prism 7 software (GraphPad, San Diego, CA, USA).

## Results

### *SMAD2* is upregulated in NSCLC tissues and cell lines

**Figure 1:**
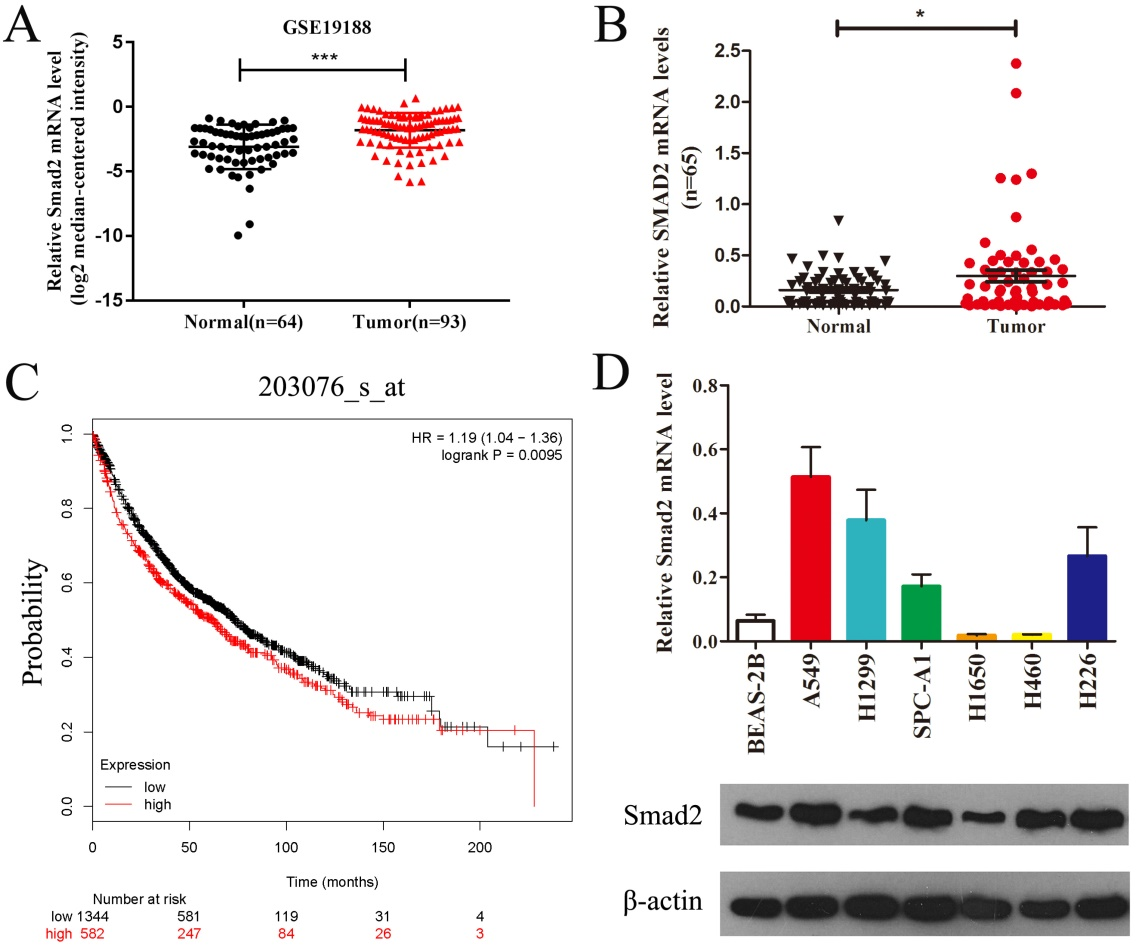
** SMAD2 is upregulated in NSCLC tissues and cell lines. (A)** Data obtained from GEO (GSE19188) were analyzed to compare the expression difference of SMAD2 between NSCLC tissues and noncancerous lung tissues. **(B)** The mRNA expression levels of *SMAD2* were determined by qRT-PCR, and the data were compared between 65 NSCLC and paired adjacent noncancerous lung tissues.**(C)** The relationship between the expression levels of SMAD2 and overall survival for 1926 NSCLC patients were analyzed, and Kaplan-Meier plots were generated using the Kaplan-Meier Plotter (http://www.kmplot.com).**(D)** Total RNA and protein were extracted from several cell lines, and the levels of SMAD2 mRNA and protein were detected by qRT-PCR and western blotting assay, respectively. β-actin was used as an internal control. Data are shown as the mean ± SE. *, **, and *** indicate significant differences compared with the control (* *P*<0.05; ** P < 0.01; ***P<0.001).

Public data from Gene Expression Omnibus (GSE19188) showed that *SMAD2* mRNA expression was significantly upregulated in lung carcinoma compared with that in normal lung tissues (Figure 1A). We verified the expression of *SMAD2* mRNA in 65 paired NSCLC tissues and adjacent noncancerous lung tissues and found that *SMAD2* mRNA levels were significantly higher in NSCLC tissues than in the adjacent noncancerous lung tissues (*P* < 0.05, Figure 1B and Table 1). Patient characteristics with respect to the decreased expression of SMAD2 and miR-486-5p are shown in Table 1. No significant difference in the SMAD2 and miR-486-5p mRNA level was observed in the NSCLC samples age, gender, histology, lymph node status, smoking history and distant metastases. In addition, the public dataset from Kaplan-Meier Plotter (http://www.kmplot.com) indicated that high expression of SMAD2 was significantly associated with poor survival of patients with NSCLC (*P* < 0.01, Figure 1C). Next, we detected SMAD2 mRNA and protein levels in six NSCLC cell lines and in a bronchial epithelial cell line, BEAS-2B, using qRT-PCR and western blotting analysis (Figure 1D). The results showed that the SMAD2 levels in the NSCLC cell lines were higher than those in the BEAS-2B cells. Collectively, our data showed that SMAD2 is upregulated in NSCLC tissues and cell lines.

### Knockdown of *SMAD2* inhibits cell EMT and the migration and invasion of NSCLC cells

**Figure 2:**
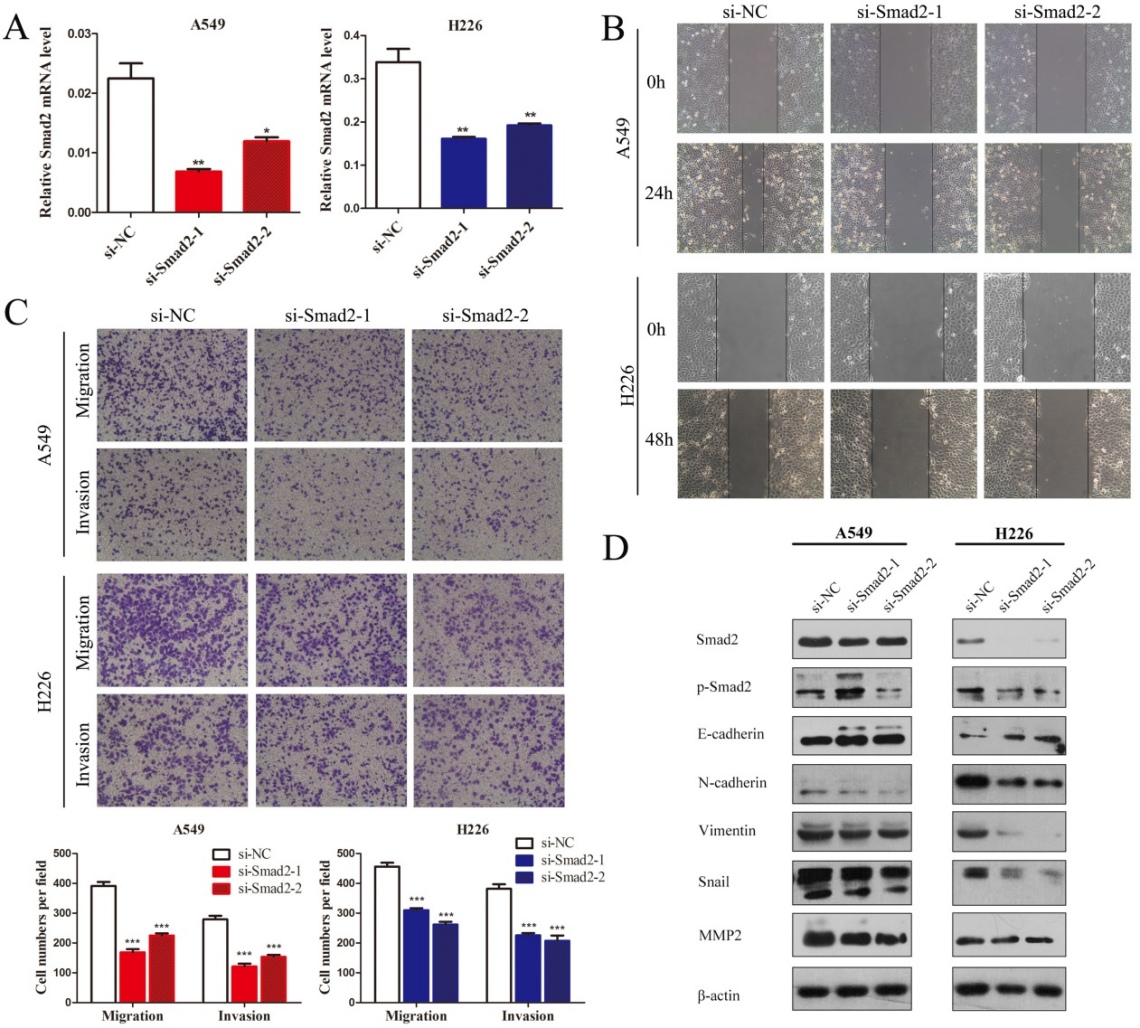
** Knockdown of SMAD2 inhibits cell EMT and the migration and invasion of NSCLC cells. (A)** Si-RNAs against *SMAD2* were transfected into A549 (left) and H226 (right) cells for 48 h. The cells were subjected to determination of *SMAD2* mRNA expression using qRT-PCR. **(B)** A uniform scratch was made in each confluent monolayer of A549 (upper) cells and H226 (bottom) cells transfected with siRNAs for *SMAD2*. Images were acquired at 0 h and 24 h post scratching under a microscope. **(C)** A549 and H226 cells knocked down for *SMAD2* were allowed to migrate through an 8-µm pore in a Transwell apparatus. Migrated cells were stained and counted in at least three microscopic fields 24 h later. One representative image (upper) is shown, and the number of migrated cells was compared between groups of SMAD2-silenced and negative control (bottom). After transfection with *SMAD2* siRNAs for 48 h, A549 and H226 cells were subjected to western blotting to determine the expression of various proteins. β -actin was used as an internal control. Data are shown as the mean ± SD. *, **, and *** indicate significant differences compared with the control (* *P* < 0.05; ** *P* < 0.01; ***P < 0.001).

SMAD2 is an important element of the TGF-β signaling pathway that plays a critical role in cell EMT and invasion. Hence, we first observed the influence of knockdown of *SMAD2* on the cell migration and invasion of NSCLC cells. Two siRNAs were used to inhibit *SMAD2* expression in A549 and H226 cells (Figure 2A). Wound healing tests showed that knockdown of *SMAD2* markedly inhibited the migratory capacity of A549 and H226 cells (Figure 2B). As shown in Figure 2C, in Transwell assays, cell migration and invasion were significant suppressed in both A549 and H226 cells after knockdown of *SMAD2*. Moreover, EMT was inhibited by knockdown of *SMAD2* in the NSCLC cells, which was manifested as downregulated levels of p-SMAD2, N-cadherin, Vimentin, Snail, and matrix metalloproteinase 2 (MMP2), and upregulated E-cadherin, compared with their levels in the negative control cells (Figure 2D). Taken together, the results showed that SMAD2 is involved in EMT and invasion of NSCLC cells.

### Knockdown of *SMAD2* represses TGF-β-induced cell EMT, and migration and invasion in NSCLC cells

**Figure 3:**
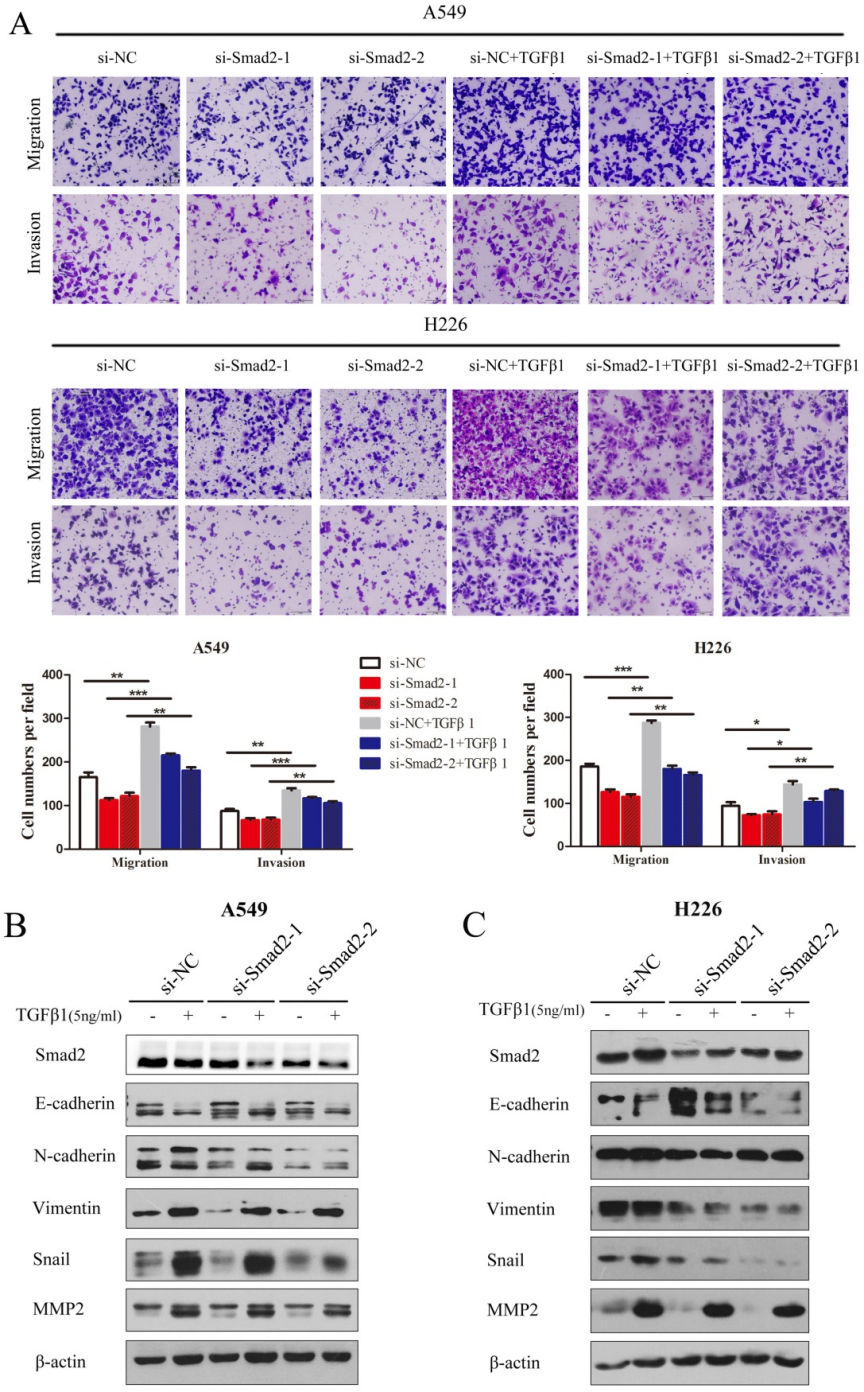
** Knockdown of *SMAD2* represses TGF-β-induced EMT, and migration and invasion in NSCLC cells. (A)** In the presence or absence of TGF-β1 (5 ng/mL), A549 and H226 cells knocked down for *SMAD2* were allowed to migrate through an 8-µm pore in a Transwell apparatus. One day later, migrated cells were stained and counted in at least three microscopic fields. One representative image (upper) is shown, and the number of migrated cells was compared between groups of si-SMAD2 and si-NC in the presence of TGF-β1 (bottom).**(B and C)** A549 (B) and H226 (C) cells transfected with siRNAs for SMAD2 or si-NC were treated with TGF-β1 (5 ng/mL) for 24 h, and then the expression of various proteins were determined by western blotting. β-actin was used as an internal control. Data are shown as the mean ± SD. *, **, and *** indicate significant differences compared with the control (* *P* < 0.05; ** *P* < 0.01; ***P < 0.001).

TGF-β1 was introduced and cell migration and invasion were determined. TGF-β induced the migration and invasion of A549 cells, which was inhibited by knockdown of *SMAD2* (Figure 3A, upper panel). The results were similar in H226 cells (Figure 3A, lower panel). TGF-β-induced EMT was estimated by detecting several EMT markers that could be regulated by TGF-β. As shown in Figure 3B and C, treatment with TGF-β1 caused levels of E-cadherin to decrease, while N-cadherin, Vimentin, Snail, and MMP2 levels increased in A549 (Figure 3B) and H226 cells (Figure 3C). However, the TGF-β1-induced variation in EMT maker levels was inhibited by siRNA-mediated knockdown of *SMAD2* in A549 (Figure 3B) and H226 cells (Figure 3C). These results showed that knockdown of *SMAD2* expression repressed TGF-β1-induced EMT in NSCLC cells.

### MiR-486-5p inhibits *SMAD2* expression by 3' UTR-binding, and is downregulated in NSCLC tissues and cell lines

**Figure 4:**
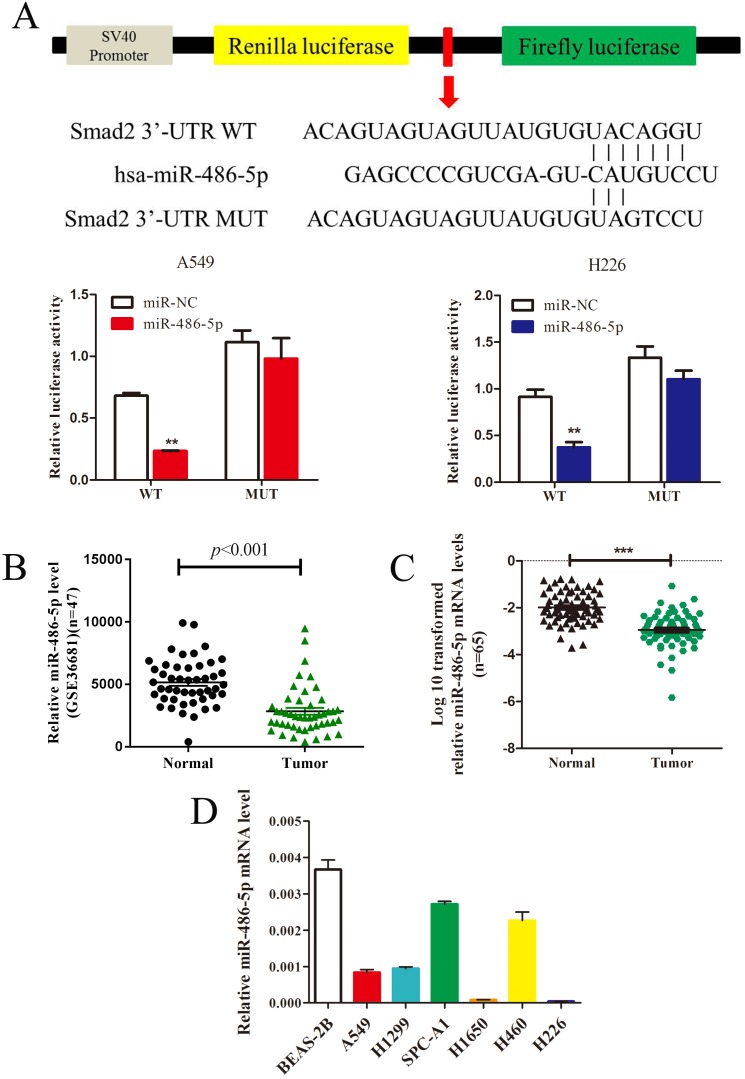
** MiR-486-5p inhibits SMAD2 expression via binding to its 3**' **UTR and is downregulated in NSCLC tissues and cell lines. (A)** Schematic diagram showing the subcloning of the predicted miR-486-5p-binding site at position 304-310 of the *SMAD2* 3' UTR into a psiCHECK-2 luciferase construct. Predicted duplex formation between miR-486-5p and the wild-type or mutant miR-486-5p-binding site is indicated (upper). Luciferase activity of the construct containing the wild-type or mutant *SMAD2* reporter gene in A549 (bottom, left) and H226 (bottom, right) cells co-transfected with the negative control (miR-NC) or miR-486-5p. Scrambled sequences were used as the NC. Relative Renilla luciferase activity was determined and normalized against firefly luciferase activity. **(B)** Data obtained from the GEO database (GSE36681) were analyzed to compare the expression of miR-486-5p between NSCLC tissues and noncancerous lung tissues.** (C)** The mRNA expression levels of miR-486-5p were determined using qRT-PCR, and the data were compared between 65 NSCLC and paired adjacent noncancerous lung tissues. **(D)** qRT-PCR analysis of relative miR-486-5p expression in human NSCLC cell lines. Data are shown as the mean ± SD. *, **, and *** indicate significant differences compared with the control (* *P* < 0.05; ** *P* < 0.01; ***P < 0.001).

*In silico* prediction (TargetScan) showed that *SMAD2* is a potential target of miR-486-5p. To verify this, a dual-luciferase reporter vector containing the *SMAD2* 3' UTR seed region specific to miR-486-5p, or the corresponding mutant sequence, was used. As shown in Figure 4A, the luciferase activities of the reporter vector containing the seed region of miR-486-5p in A549 and H226 cells was significantly suppressed by miR-486-5p transfection compared with that in the cells transfected with miR-NC. However, the activities were restored when the seed region was mutated. Considering the increased expression of *SMAD2* in NSCLC tissues and cell lines, we hypothesized that miR-486-5p might be decreased in NSCLC. As expected, the GEO data (GSE36681) showed that the expression of miR-486-5p was downregulated in NSCLC tissues. We also detected that the expression level of miR-486-5p was significantly decreased in 65 NSCLC tissues relative to their paired noncancerous tissues; these tissues were collected and stored by our research group. Consistent with the tissue expression data, miR-486-5p was also downregulated in several NSCLC cell lines compared with its level in BEAS-2B cells.

### MiR-486-5p represses *SMAD2* expression, and the capability of migration and invasion of NSCLC cells

**Figure 5:**
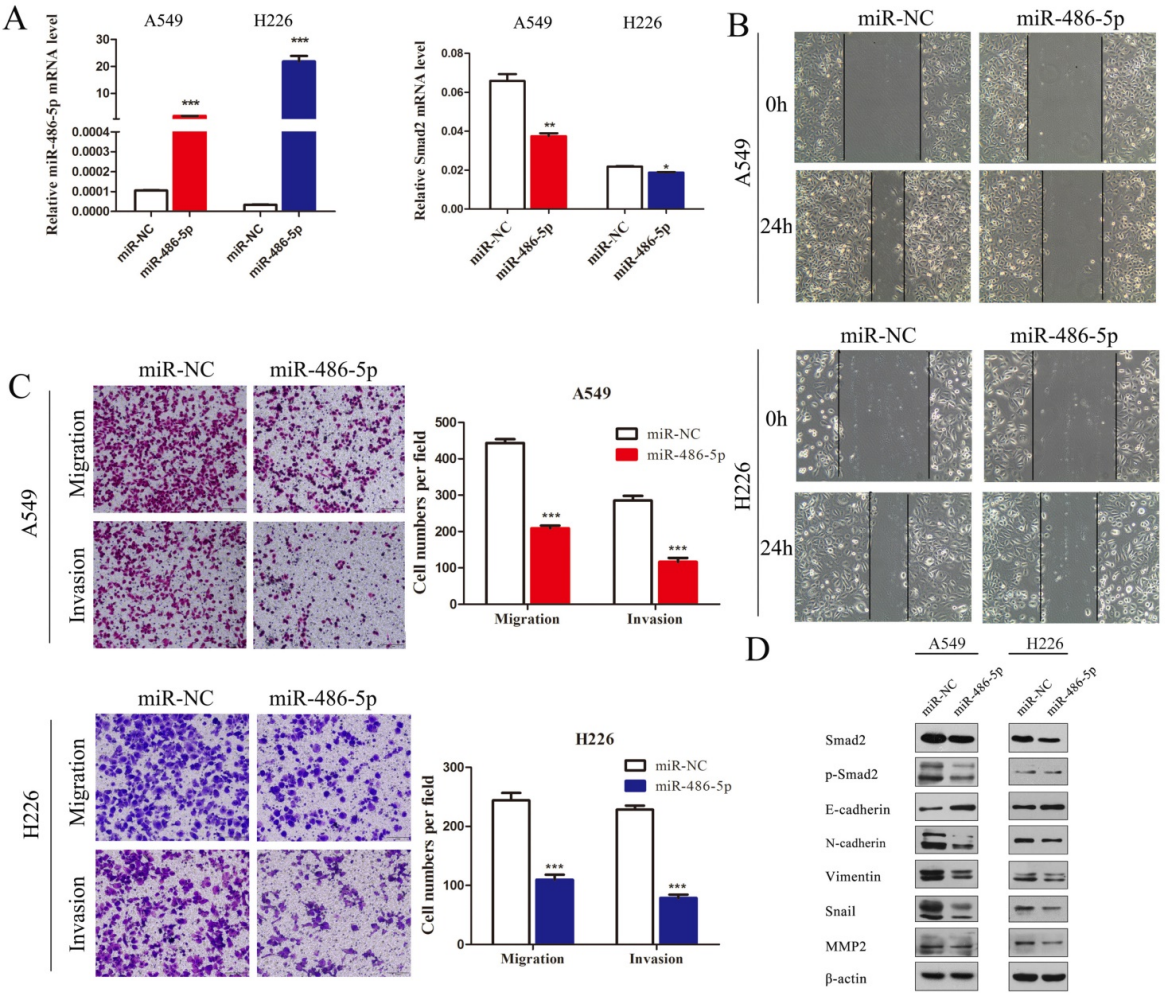
** MiR-486-5p represses SMAD2 expression, and the migration and invasion abilities of NSCLC cells. (A)** miR-486-5p mimics were transfected into A549 and H226 cells for 48 h. The expression levels of miR-486-5p (left) and SMAD2 mRNA (right) were determined in the cells using qRT-PCR assay. **(B)** A uniform scratch was made in each confluent monolayer of A549 (upper) cells and H226 (bottom) cells transfected with miR-486-5p or miR-NC. Images were acquired at 0 h and 24 h post scratching under a microscope. **(C)** A549 and H226 cells transfected with miR-486-5p or miR-NC were allowed to migrate through an 8-µm pore in a Transwell apparatus. Migrated cells were stained and counted in at least three microscopic fields 24 h later. One representative image (left) is shown, and the number of migrated cells was compared between the miR-486-5p and miR-NC groups (right). After transfection with miR-486-5p for 48 h, A549 and H226 cells were subjected to western blotting to determine the expression of various proteins. β-actin was used as an internal control. Data are shown as the mean ± SD. *, **, and *** indicate significant differences compared with the control (* *P* < 0.05; ** *P* < 0.01; ***P < 0.001).

**Figure 6:**
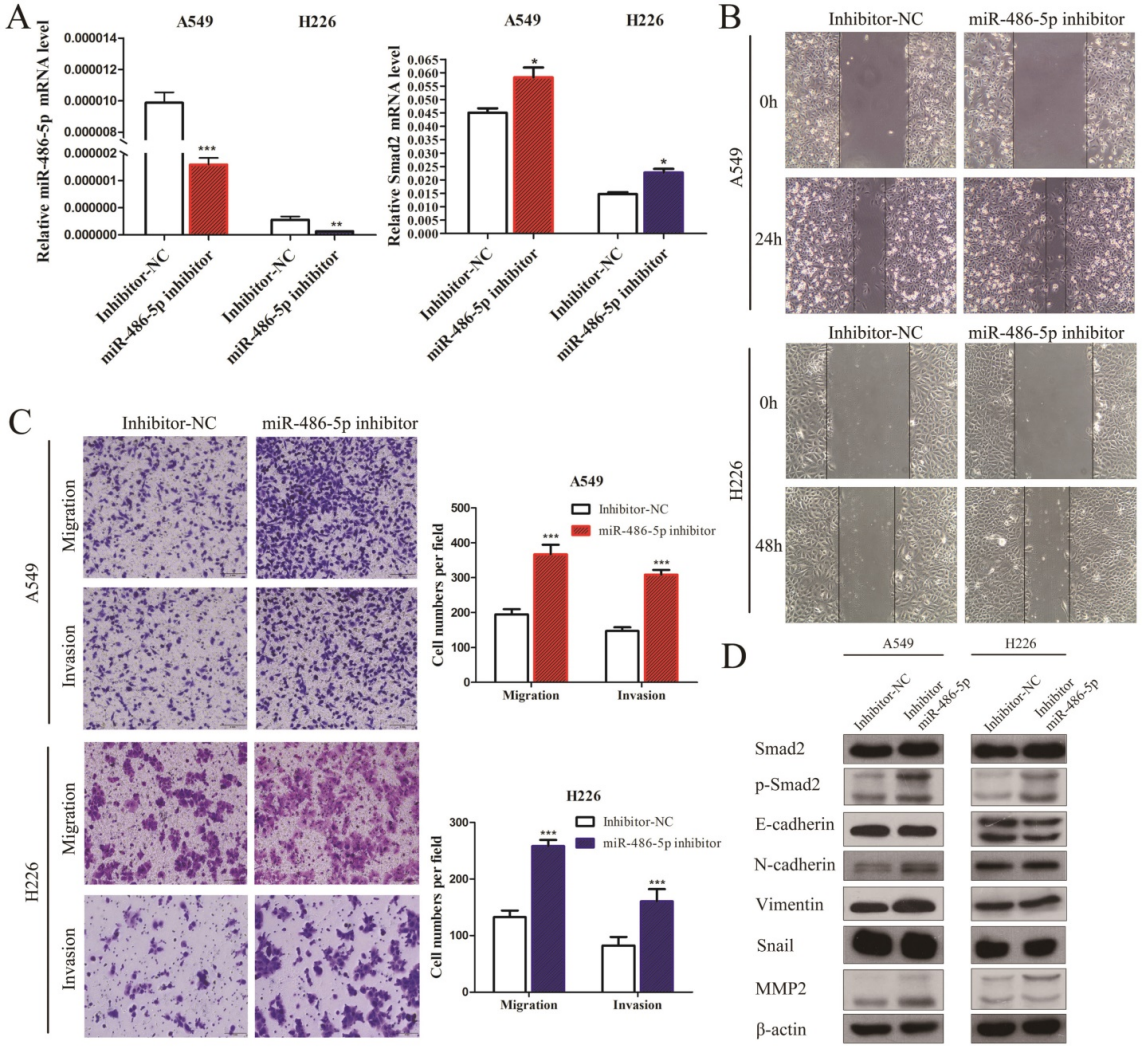
** MiR-486-5p inhibitor enhances SMAD2 expression, and the capability of migration and invasion of NSCLC cells. (A)** The miR-486-5p inhibitor mimics or negative control (inhibitor-NC) were transfected into A549 and H226 cells for 48 h. The expression levels of miR-486-5p (left) and *SMAD2* mRNA (right) were then determined using qRT-PCR. **(B)** A uniform scratch was made in confluent monolayers of A549 (upper) cells and H226 (bottom) cells the transfected of the miR-486-5p inhibitor or inhibitor-NC. Images were acquired at 0 h and 24 h post scratching under a microscope. **(C)** A549 and H226 cells transfected with the miR-486-5p inhibitor or inhibitor-NC were allowed to migrate through an 8-µm pore in a Transwell apparatus. Migrated cells were stained and counted in at least three microscopic fields 24 h later. One representative image (left) is shown, and the number of migrated cells was compared between the miR-486-5p inhibitor and inhibitor-NC groups (right). After transfection with the miR-486-5p inhibitor for 48 h, A549 and H226 cells were subjected to western blotting to determine the expression of various proteins. β-actin was used as an internal control. Data are shown as the mean ± SD. *, **, and *** indicate significant differences compared with the control (* *P* < 0.05; ** *P* < 0.01; ***P < 0.001).

A549 and H226 cells transiently transfected with miR-486-5p or the negative control (miR-NC) (Figure 5A, left panel) were subjected cell migration and invasion assays, and their expression of EMT markers was detected. *SMAD2* mRNA expression was suppressed by miR-486-5p transfection in A549 and H226 cells (Figure 5A, right panel). Wound healing assays showed that cells transfected with miR-486-5p migrated slower than the control cells (Figure 5B). Similarly, Transwell assays showed that miR-486-5p inhibited the cell migration and invasion capability of A549 and H226 cells (Figure 5C). The different levels of several EMT markers related to cell migration were determined between the cells overexpressing miR-486-5p and miR-NC using western blotting. The results showed that overexpression of miR-486-5p in A549 and H226 cells significantly suppressed the protein levels of SMAD2 and phosphorylation of SMAD2 (p-SMAD2), and inhibited the EMT process, as reflected by the upregulation of E-cadherin and the downregulation of N-cadherin, Vimentin, Snail, and MMP2 levels. These results were in turn confirmed by transfection with miR-486-5p inhibitor mimics. As shown in Figure 6A, transfection of the miR-486-5p inhibitor mimics in A549 and H226 cells markedly inhibited the expression of miR-486-5p (left panel), and relieved the inhibition on* SMAD2* mRNA expression induced by miR-486-5p (right panel). In contrast to miR-486-5p, the inhibitor mimics dramatically promoted cell migration and invasion, as determined by wound healing (Figure 6B) and Transwell assays (Figure 6C) in A549 and H226 cells. Meanwhile, inhibition of miR-486-5p significantly increased total SMAD2 and p-SMAD2 levels, and promoted the EMT process, as demonstrated by downregulation of E-cadherin and upregulation of N-cadherin, Vimentin, Snail, and MMP2 levels (Figure 6D). Taken together, these results showed that miR-486-5p inhibits SMAD2 activity, cell EMT, and cell migration in NSCLC.

### MiR-486-5p inhibits TGF-β-induced cell EMT, and migration and invasion in NSCLC cells

**Figure 7:**
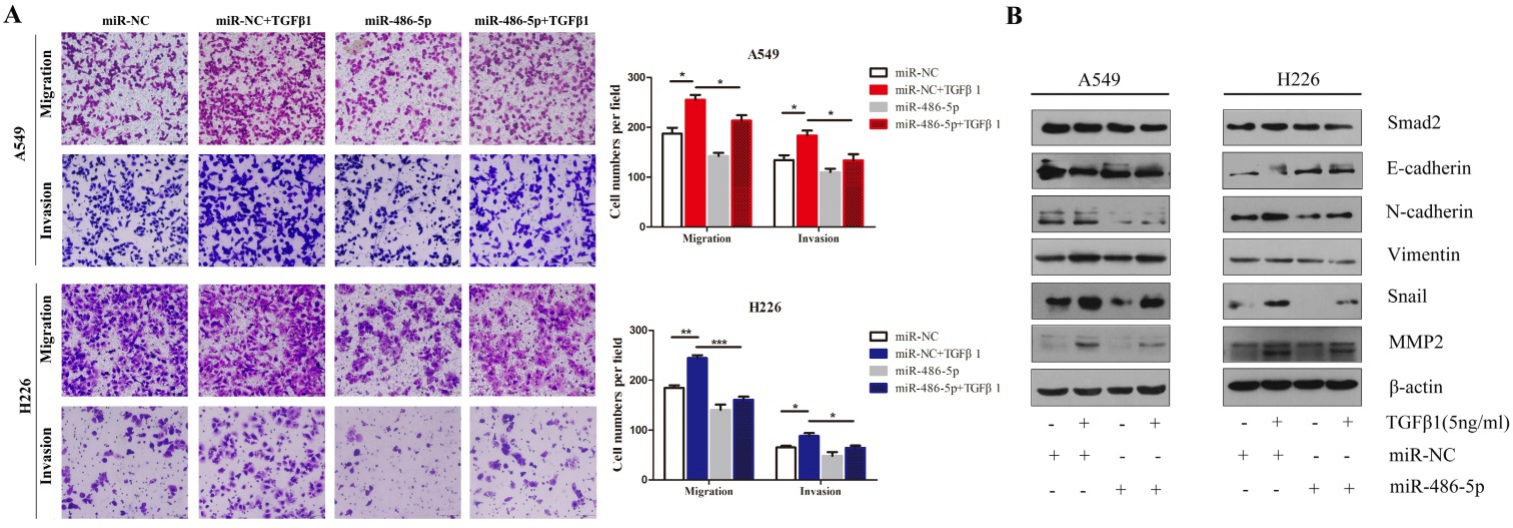
** MiR-486-5p inhibits TGF-β-induced cell EMT, and migration and invasion in NSCLC cells.** (A) In the presence or absence of TGF-β1 (5 ng/mL), A549 and H226 cells transfected with miR-486-5p were allowed to migrate through an 8-µm pore in a Transwell apparatus. One day later, migrated cells were stained and counted in at least three microscopic fields. One representative image (upper) is shown, and the number of migrated cells was compared between the miR-486-5p and miR-NC groups in the presence of TGF-β1 (bottom). (B and C) A549 (B) and H226 (C) cells transfected with miR-486-5p or miR-NC were treated with TGF-β1 (5 ng/mL) for 24 h, and then the expression of various proteins was determined by western blotting. β-actin was used as an internal control. Data are shown as the mean ± SD. *, **, and *** indicate significant differences compared with the control (* *P* < 0.05; ** *P* < 0.01; ***P < 0.001).

Considering that SMAD2 is implicated in TGF-β-induced cell EMT, we hypothesized that miR-486-5p would influence TGF-β-induced cell EMT and cell migration. As shown in Figure 7A, miR-486-5p significantly suppressed cell migration and invasion in both A549 and H226 cells in the presence of TGF-β1 (Figure 7A). In addition H226 cells, the variation in the levels of EMTmarkers - downregulation of E-cadherin, upregulation EMT markers of N-cadherin, Vimentin, Snail, and MMP2 - were prevented by miR-486-5p overexpression in A549 and H226 cells (Figure 7B). These data are in line with the effect of knockdown of *SMAD2* on TGF-β-induced EMT, suggesting an EMT-repressive role of miR-486-5p in NSCLC cells.

## Discussion

Metastasis is the main reason for cancer related death from solid tumors, including NSCLC. The EMT process contributes markedly to the progress of tumor metastasis. The TGF-β/SMADs signaling plays an essential role in accelerating EMT and cancer cell migration. As a member of the SMAD family, SMAD2 plays a key role, together with SMAD3 and SMAD4, in TGF-β signaling and cancer progression 25. In the present study, we found that SMAD2expression was upregulated in NSCLC tissues and cell lines, and that high levels of *SMAD2*mRNA were associated with poor prognosis of NSCLC, exhibiting an oncogenic potency in NSCLC. A previous study showed that silencing* SMAD2* restrained TGF-β-induced EMT andcell invasion, indicating a therapeutic role for SMAD2 inhibition in cancer 26. MiRNAs are small interfering RNA (siRNAs) with a length of about 20 bp that are produced in organisms. Not only can one miRNA can inhibit the expression of multiple genes, but also one gene can be targeted by multiple miRNAs. SMAD2 has been proven to be targeted and downregulated by various miRNAs such as miR-132 27, miR-27a 28, and miR-323-3p 29 in regulating multiple cancers.

We observed an interaction between SMAD2 and miRNAs, such that SMAD2 expression was suppressed by the direct binding of miR-486-5p to the 3' UTR of *SMAD2*, providing a new insight into developing a therapeutic strategy for NSCLC. The interaction also suggested that miR-486-5p might have a great impact on cell EMT and migration in NSCLC. These results led to a similar conclusion to a recent study in lens epithelial cells that miR-486-5p prevents cell migration and invasion by targeting SMAD2 30. Moreover, a study in prostate cancer showed that miR-486-5p suppresses metastasis by targeting Snail and regulating the EMT process 31. We found that miR-486-5p repressed TGF-β-induced EMT and cell migration and invasion in NSCLC cells.

Consistent with these proposed functions, we found that miR-486-5p expression was downregulated in NSCLC, which further confirmed a tumor suppressor role of miR-486-5p in cancer. Shao et al. recently reported that miR-486-5p is downregulated in NSCLCs and suppresses cell proliferation by direct inhibiting the expression of the oncogene CDK4^32^. MiR-486-5p is supported in its activities as a tumor suppressor in lung cancer by regulating insulin growth factor (IGF) signaling 33. In studies on other cancers, miR-486-5p expression was decreased in gastric adenocarcinomas, esophageal squamous cell carcinomas, and colorectal carcinomas 34, 35. It is not unusual that one miRNA performs different functions in different cancer types. For instance, miR-486-5p is overexpressed in chronic myeloid leukemia and can enhance cell growth via regulation of AKT signaling and FOXO1 expression^36^.

In conclusion, in the present study, SMAD2, as an important member of the TGF-β signaling pathway, was proven to be targeted and downregulated by miR-486-5p in NSCLC cells. MiR-486-5p expression is downregulated in NSCLCs, whereas SMAD2 is upregulated. Overexpression of miR-486-5p prevented TGF-β-induced EMT and cell migration and invasion in NSCLC cells. Our study provides new insights into the theoretical basis of NSCLC and therapeutic strategies that may be used to treat it.

## Figures and Tables

**Table 1:** Demographic and clinical characteristics of NSCLC patients and the level of miR-486-5p and Smad2 mRNA expression in tumor tissue specimens

Characteristics	Number of cases (%)	miR-486-5p	*P* value	Smad2 mRNA	*P* value
**Age (years)**					
≤65	30(46.2%)	0.0034 ± 0.0010	0.756	0.1888 ± 0.0505	0.070
>65	35(53.8%)	0.0042 ± 0.0023		0.3947 ± 0.0938	
**Gender**					
Male	42(64.6%)	0.0046 ± 0.0021	0.467	0.3690± 0.0838	0.098
Female	23(35.4%)	0.0025 ± 0.0006		0.1730 ± 0.0374	
**Histological features**					
Adenocarcinoma	34(52.3%)	0.0032 ± 0.0009	0.142	0.2905 ± 0.0607	0.967
Squamous cell carcinoma	25(38.5%)	0.0054 ± 0.0033		0.3178 ± 0.1227	
Others	6(9.2%)	0.0007 ± 0.0003		0.2762 ± 0.0789	
**Degree of differentiation**					
Low	25(38.5%)	0.0017 ± 0.0004	0.211	0.2271 ± 0.0619	0.315
Middle	40(61.5%)	0.0052± 0.0022		0.3451 ± 0.0835	
**Smoker**					
Yes	34(52.3%)	0.0053 ± 0.0025	0.274	0.3461 ± 0.0948	0.395
No	31(47.6%)	0.0023 ± 0.0005		0.2487 ± 0.0581	
**Clinical stage**					
I +II	33(50.8%)	0.0051 ± 0.0025	0.359	0.2653 ± 0.0730	0.543
III + IV	32(49.2%)	0.0026 ± 0.0008		0.3351 ± 0.0880	
**Distant metastasis**					
No	56(86.2%)	0.0040 ± 0.0015	0.744	0.2571± 0.0576	0.060
Yes	9(13.8%)	0.0027 ± 0.0017		0.5649± 0.1833	
**Lymph node metastasis**					
No	35(53.8%)	0.0052 ± 0.0024	0.283	0.3509 ± 0.0841	0.334
Yes	30(46.2%)	0.0022 ± 0.0008		0.2399 ± 0.0740	

Data are presented as mean ± SEM. Kruskal-Wallis test for comparison between three or more groups.
